# Comparative Effects of Different Statin Regimens on Postoperative Outcomes in Isolated Coronary Artery Bypass Grafting: A Propensity Score-Matched Analysis

**DOI:** 10.3390/jcm15156060

**Published:** 2026-08-04

**Authors:** Osman Fehmi Beyazal, Zeki Temizturk, Cemalettin Aydin, Mehmet Okan Donbaloglu, Arjen Erdal, Selman Sadi Citak, Nihan Kayalar, Mehmed Yanartas

**Affiliations:** Department of Cardiovascular Surgery, Basaksehir Cam and Sakura City Hospital, Istanbul 34480, Turkey; zekitmztrk5806@gmail.com (Z.T.); cemalettinaydin1@hotmail.com (C.A.); donbalogluokan@hotmail.com (M.O.D.); arjenerdal72@gmail.com (A.E.); dr.selmansadi@gmail.com (S.S.C.); nkayalar@hotmail.com (N.K.); myanartas@yahoo.com (M.Y.)

**Keywords:** coronary artery bypass grafting, atrial fibrillation, atorvastatin, rosuvastatin, acute kidney injury

## Abstract

**Background:** This study aimed to evaluate the impact of various preoperative statin types and dosage regimens on early postoperative outcomes following isolated coronary artery bypass grafting (CABG). **Methods:** A total of 950 patients who underwent isolated CABG between 2022 and 2026 were evaluated and stratified into three groups based on their preoperative statin status: Group A, statin-naive patients (*n =* 592); Group B, atorvastatin users (*n =* 293); and Group C, rosuvastatin users (*n =* 65). To analyze the dose-dependent outcomes of statins, patients in Group B were subsequently segregated into two subgroups and matched using propensity score matching: Group 3, low-to-moderate regimen (≤20 mg/day; *n =* 75), and Group 4, high-dose regimen (>20 mg/day; *n =* 75). **Results**: Except for the higher incidence of diabetes mellitus and hypertension in statin-treated patients, all pre- and intraoperative characteristics of Groups A, B, and C were similar, and all of their postoperative outcomes except new-onset atrial fibrillation (NOAF) were similar. NOAF incidence was significantly higher in statin-naive patients compared to both the atorvastatin and rosuvastatin cohorts [109 (18.4%) - 35 (11.9%) - 7 (10.8%), *p* = 0.02]. All early postoperative outcomes—including in-hospital mortality and NOAF incidence—were found to be fully similar between Groups 3 and 4. Furthermore, multivariable analysis confirmed preoperative statin use as an independent protective factor against NOAF. **Conclusions:** The preoperative utilization of either atorvastatin or rosuvastatin was associated with a lower NOAF incidence in isolated CABG, and no significant differences were observed between the atorvastatin and rosuvastatin cohorts regarding NOAF and other postoperative outcomes. Preoperative atorvastatin administration at doses below and above 20 mg/day showed no significant differences in terms of postoperative outcomes.

## 1. Introduction

Hydroxymethylglutaryl-coenzyme A reductase inhibitors (statins) hold a significant place in cardiovascular disease treatment. According to the 2023 European Society of Cardiology (ESC) Guidelines for the Management of Acute Coronary Syndromes, administering and continuing high-dose statin therapy is recommended as early as possible, regardless of the initial low-density lipoprotein cholesterol (LDL-C) levels [1]. However, beyond their primary lipid-lowering actions, statins also possess notable antithrombotic and pleiotropic effects [2].

Although some studies have reported that statins reduce postoperative atrial fibrillation (POAF) and acute kidney injury (AKI) after cardiac surgery, others have indicated that AKI can be more common compared to placebo groups and that the risk of POAF does not decrease [3]. Consequently, whether statins should be routinely used in the preoperative period remains unclear [4]. In our previous study, we also found that statin use was protective against POAF [5]. However, a major limitation in studies on this topic in cardiac surgery, including our own, is the absence of a detailed comparison regarding specific statin types and doses. It remains uncertain whether different medications frequently used in coronary artery bypass grafting (CABG) patients, such as atorvastatin and rosuvastatin, or various dosage regimens are superior to each other regarding postoperative outcomes. Despite numerous studies, it is still unclear whether preoperative statins should be used in CABG patients, and if so, which statin should be used and at what dose. Therefore, the purpose of this study was to investigate whether different preoperative statin types and dosages have an effect on postoperative outcomes in patients undergoing isolated CABG.

## 2. Methods

This investigation was structured as a single-center, retrospective, and observational cohort study. Clinical records of 950 consecutive patients who underwent isolated CABG at the Department of Cardiovascular Surgery of Istanbul Basaksehir Cam and Sakura City Hospital between January 2022 and June 2026 were fully screened for eligibility. To establish a homogeneous study population, specific exclusion criteria were strictly applied; emergency procedures, patients undergoing concomitant procedures, cases requiring postoperative extracorporeal membrane oxygenation (ECMO) support, and individuals receiving preoperative lipid-lowering agents other than atorvastatin or rosuvastatin were excluded from the final analysis. All operations were performed by the same surgical team throughout the study period. The study population was stratified into three categories based on their preoperative statin status: Group A comprised patients with no history of statin administration (*n =* 592), Group B encompassed those under atorvastatin treatment (*n =* 293), and Group C consisted of individuals utilizing rosuvastatin (*n =* 65). To analyze the dose-dependent outcomes of statin therapy on postoperative outcomes, patients within the atorvastatin cohort (Group B) were subsequently segregated into two subgroups: Group 1, low-to-moderate regimen (≤20 mg/day; *n =* 171), and Group 2, high-dose regimen (>20 mg/day; *n =* 122). To effectively eliminate potential confounding factors arising from baseline clinical disparities between these two dosage subgroups, a 1:1 propensity score-matched (PSM) analysis was performed. The matching model adjusted for critical perioperative variables, including cardiopulmonary bypass (CPB) times, cross-clamp (XCL) times, the total number of distal anastomoses, patient age, sex, preoperative left ventricular ejection fraction (EF), diabetes mellitus (DM), and hypertension (HT). Following this matching process, two new balanced subgroups were obtained: Group 3 (atorvastatin ≤ 20 mg/day; *n =* 75) and Group 4 (atorvastatin > 20 mg/day; *n =* 75).

Institutional electronic databases and physical charts were comprehensively reviewed to extract detailed medical profiles for all participants. The compiled dataset encompassed baseline demographic characteristics, specific statin types and exact dosing strategies, chronic comorbidities, preoperative transthoracic echocardiographic measurements, laboratory parameters, and the European System for Cardiac Operative Risk Evaluation (EuroSCORE) II values. Additionally, detailed intraoperative data consisting of specific surgical techniques, CPB times, and XCL times were documented. The prespecified primary endpoint of this study was the incidence of postoperative new-onset atrial fibrillation (NOAF). Secondary exploratory endpoints included a broad spectrum of early postoperative complications, namely total postoperative mediastinal bleeding, cerebrovascular accidents (CVAs), requirements for continuous renal replacement therapy (CRRT), deep sternal wound infections, gastrointestinal bleeding, percutaneous coronary interventions (PCIs), major adverse cardiac and cerebrovascular events (MACCEs), all-cause mortality, vasoactive inotropic score (VIS), intubation times, and the length of stay in the intensive care unit (ICU) and hospital.

The study protocol received formal approval from the Istanbul Basaksehir Cam and Sakura City Hospital Ethics Committee (Decision no: 2025-319, Date: 17 June 2026), and it was conducted in accordance with the Declaration of Helsinki. No artificial intelligence-assisted technologies or generative language models were utilized in the drafting, analysis, or production of the submitted manuscript.

## 3. Statistics

All statistical evaluations were carried out using IBM SPSS Statistics software (version 27.0; IBM Corp., Armonk, NY, USA). Continuous variables are reported as median values with corresponding interquartile ranges (IQRs), along with minimum and maximum values where appropriate, whereas categorical variables are presented as counts and percentages. The distribution of continuous data was assessed using the Kolmogorov–Smirnov test. Based on the normality of the distribution, comparisons across the study groups were performed using the independent-samples t-test, Mann–Whitney U test, or Kruskal–Wallis test. For categorical data, either the Pearson chi-square test or Fisher’s exact test was applied, depending on the expected cell counts. PSM was applied to reduce potential confounding effects between the atorvastatin dosage subgroups. In this study, matching was performed using a 1:1 nearest-neighbor method, without replacement, and with a 0.2 SD caliper based on the logit of the propensity score. Observations outside of common support were excluded to increase comparability between the two groups. Matching quality was evaluated using the standardized mean difference (SMD), and multivariate logistic regression analysis was performed to determine the factors affecting NOAF. A two-sided *p*-value of less than 0.05 was considered indicative of statistical significance. There were no missing observations for any of the primary and secondary clinical endpoints, nor for the covariates incorporated into the propensity score matching and multivariable logistic regression models; therefore, a complete-case analysis was performed for these evaluations.

## 4. Results

A comprehensive comparison of baseline demographics, clinical comorbidities, echocardiographic parameters, and operative data across Groups A, B, and C is illustrated in Table 1. The mean age of the entire study population was 60.2 ± 9.1 years, and 778 patients (81.8%) were male. Statistical evaluation revealed that patient age, sex distribution, height, and weight profiles were fully comparable among the cohorts. Chronic comorbidities, specifically DM and HT, were significantly more prevalent in both the atorvastatin and rosuvastatin cohorts than in the statin-naive group [DM: 309 (52.2%) vs. 181 (61.8%) vs. 40 (61.5%), *p* = 0.01; HT: 327 (55.2%) vs. 204 (69.6%) vs. 47 (72.3%), *p* < 0.001]. Regarding other concurrent clinical comorbidities, no significant discrepancies were detected between the groups. The baseline EuroSCORE II parameters also demonstrated equity across the cohorts, with respective medians of 1.3, 1.3, and 1.4 (*p* = 0.88). Transthoracic echocardiographic assessments, including left ventricular EF and tricuspid annular plane systolic excursion (TAPSE) monitored preoperatively and at the postoperative 1st week, exhibited no statistical divergence. Intraoperatively, XCL and CPB times showed no statistically significant differences among the three arms [XCL medians: 78 min vs. 71 min vs. 72 min, *p* = 0.18; CPB medians: 127 min vs. 119 min vs. 119 min, *p* = 0.10]. Furthermore, the median number of distal anastomoses was equivalent across the cohorts (3 vs. 3 vs. 3, *p* = 0.49). Similarly, the utilization rates of minimally invasive surgical approaches, left internal thoracic artery (LITA) usage, concurrent coronary endarterectomy, and off-pump (beating heart) surgery procedures did not differ significantly.

Intergroup comparisons of laboratory markers for Groups A, B, and C are documented in Table 2. Preoperative levels of white blood cells, hematocrit, platelets, urea, serum creatinine, sodium, potassium, alanine aminotransferase (ALT), aspartate aminotransferase (AST), and HbA1c demonstrated no significant variations among the study groups. Notably, preoperative low-density lipoprotein cholesterol (LDL-C) and total cholesterol concentrations were significantly elevated only in the statin-naive cohort compared to those receiving active atorvastatin or rosuvastatin therapy [LDL-C medians: 116 vs. 87 vs. 83 mg/dL, *p* < 0.001; total cholesterol medians: 191 vs. 159 vs. 153 mg/dL, *p* < 0.001]. Laboratory parameters evaluated at the postoperative 1st month remained comparable across all three groups, showing no statistical divergence.

**Figure 1:**
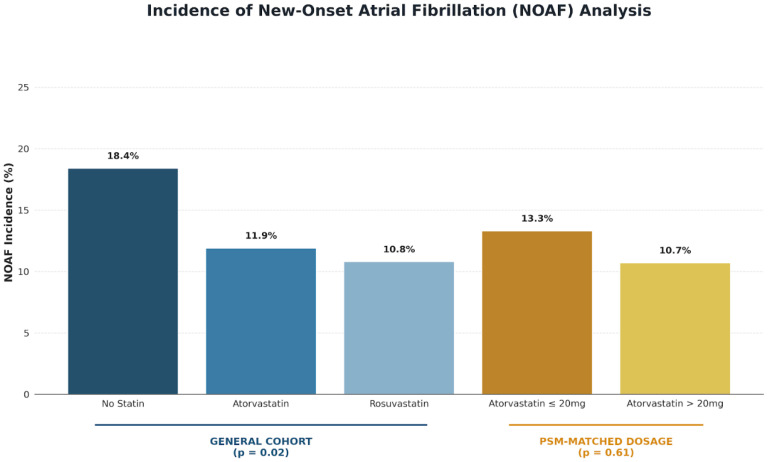
Incidence of new-onset atrial fibrillation among groups.

Early postoperative outcomes and complications for Groups A, B, and C are summarized in Table 3. CVA incidence, requirements for CRRT, deep sternal wound infections, gastrointestinal bleeding, subsequent PCI, and MACCE were statistically similar across all cohorts, and mortality rates also revealed no statistically significant variations [21 (3.5%) vs. 6 (2%) vs. 0, *p* = 0.16]. Total postoperative mediastinal bleeding volumes and maximum vasoactive inotropic scores (VISs) were statistically equivalent. No significant discrepancies were observed regarding the median intubation time (9 vs. 9 vs. 11 h, *p* = 0.07) or the length of stay in the ICU (2.2 vs. 2 vs. 2 days, *p* = 0.07). Hospital length of stay was likewise comparable among all groups (median 7 vs. 6 vs. 6 days, *p* = 0.60). However, NOAF incidence was significantly higher in statin-naive patients compared to both the atorvastatin and rosuvastatin cohorts [109 (18.4%) vs. 35 (11.9%) vs. 7 (10.8%), *p* = 0.02] (Figure 1).

A comparative evaluation of patients receiving different preoperative dosages of atorvastatin is presented in Table 4. Prior to matching, patients restricted to a standard dose (≤20 mg/day, Group 1) and those on a high-dose regimen (>20 mg/day, Group 2) displayed similar profiles regarding sex, baseline EF, and chronic comorbidities. Nevertheless, patients in Group 1 exhibited a significantly higher median age (69 vs. 59 years, *p* = 0.04) and elevated EuroSCORE II values (1.4 vs. 1.2, *p* = 0.048) than those in Group 2. While XCL times and the number of distal grafts showed no statistical differences, the median CPB times were significantly prolonged in Group 1 compared to Group 2 (122 vs. 113 min, *p* = 0.01). No significant differences were observed between Groups 1 and 2 across most postoperative outcomes, except for in-hospital mortality, which was significantly higher in the low-dose cohort [6 (3.5%) vs. 0, *p* = 0.038].

**Figure 2:**
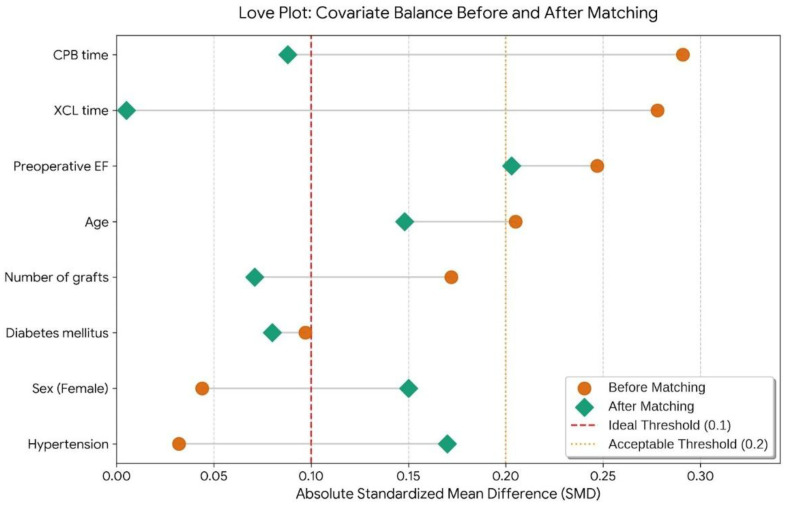
Love Plot: Covariate balance before and after matching.

To effectively eliminate these baseline imbalances, a 1:1 propensity score-matching (PSM) algorithm was executed, which successfully established a robust balance across perioperative covariates. The post-match adjustment significantly optimized covariate equity, yielding the following standardized mean difference (SMD) estimations for the primary variables: CPB duration: SMD ≈ 0.088, XCL duration: SMD ≈ 0.005, number of grafts: SMD ≈ 0.071, DM: SMD ≈ 0.080, age: SMD ≈ 0.148, sex: SMD ≈ 0.150, HT: SMD ≈ 0.170, and baseline EF: SMD ≈ 0.203. The Love Plot graph for the PSM analysis is presented in Figure 2. In the comparative analysis of the post-match subgroups (Groups 3 vs. 4), no statistically significant discrepancies were observed regarding baseline demographics, chronic comorbidities, echocardiographic outcomes, XCL times, CPB times, distal graft counts, or EuroSCORE II values. Ultimately, following the propensity-matched stabilization, all early postoperative outcomes—including in-hospital mortality and NOAF incidence—were found to be fully similar between Groups 3 and 4. 

The results of multivariate regression analysis for factors affecting NOAF are presented in Table 5. Accordingly, no significant association was found between gender, DM, HT, and NOAF, but a significant association was found between age and NOAF (*p* < 0.001, OR = 1.075 [95% CI, 0.501–1.320]). Additionally, a significant negative association was found between statin usage and NOAF (*p* = 0.01, OR = 0.609 [95% CI, 0.410–0.906]).

## 5. Discussion

Statins hold an important place in the postoperative medical management of CABG patients. Beyond coronary artery disease, their use is also recommended in peripheral arterial diseases, and it is reported that they may be protective in deep vein thrombosis [6,7]. Current evidence does not support initiating statins immediately before cardiac surgery; however, conflicts exist regarding whether patients already using statins should continue or discontinue this medication [4]. Furthermore, if statins are to be used in CABG patients, it is not entirely clear which statin and dosage should be preferred. Although we found that statins were protective against POAF in our previous study, we could not perform a comparison regarding statin types and dosages [5]. In this retrospective study, we investigated the effects of statin types and different dosage regimens on postoperative outcomes, and we observed that patients using any preoperative statin had less NOAF compared to those who did not. Additionally, in the PSM-matched atorvastatin subgroup, we determined that all postoperative outcomes, including NOAF, were similar between patients receiving doses below and above 20 mg.

Atorvastatin and rosuvastatin are among the most effective and frequently used drugs in cardiovascular diseases with similar efficacy, and atorvastatin is specifically reported to be the safest statin [8]. Both lower LDL-C levels by inhibiting hydroxymethylglutaryl-coenzyme A reductase in the cholesterol synthesis pathway [9]. Apart from this, they also possess pleiotropic, anti-inflammatory, and antioxidant effects [2,10,11]. On the other hand, these effects vary at different levels across different statins. Atorvastatin is lipophilic and can enter cells via passive diffusion, whereas rosuvastatin is hydrophilic and cannot penetrate the plasma membrane of most cells except for liver cells [12]. The majority of atorvastatin is excreted through the liver, with only 2% excreted via the kidneys, while 10% of rosuvastatin is excreted through the kidneys [13,14]. It has been reported that atorvastatin may be more beneficial than rosuvastatin regarding renal function [15]. In another meta-analysis, rosuvastatin was reported to yield better results than atorvastatin in lowering LDL-C and triglycerides and increasing high-density lipoprotein cholesterol (HDL-C) [16]. However, it has also been reported that rosuvastatin may be associated with more myopathy and acute kidney injury, especially in the elderly [17]. Many differences may cause these statins to lead to different clinical outcomes. Furthermore, different dosages may also affect clinical results. Although there are numerous studies regarding statins, there is a lack of sufficient research performing a detailed, high-volume comparison of atorvastatin and rosuvastatin in CABG patients and evaluating the effects of different dosages on postoperative outcomes.

Atorvastatin and rosuvastatin are among the most frequently used statins in clinical practice. For this reason, the vast majority of patients in our study cohort were using preoperative atorvastatin, while a relatively small portion was using rosuvastatin. Pitavastatin and other statin types were used negligibly in patients during the study period, making them statistically incomparable. Therefore, other statin types were excluded from this study. In addition, to prevent other confounders, factors with a risk of affecting postoperative outcomes were removed, and the research was planned in an isolated group. Since there are conflicts regarding the effects of preoperative statins, we did not only compare those receiving atorvastatin and rosuvastatin; patients not taking statins were also included in the analysis to evaluate both the impact of statin use and the effects of different drug types. Due to the selection of an isolated group, many factors such as demographic characteristics, echocardiographic findings, comorbidities other than DM and HT, EuroSCORE II, XCL times, and CPB times were similar across all groups in the study population. The rates of DM and HT were similar between those receiving atorvastatin and rosuvastatin, but as expected, they were higher compared to patients not taking statins. Again, as expected, LDL-C and total cholesterol levels were higher in patients not taking statins, but they were at similar levels in those taking atorvastatin and rosuvastatin. Despite the similarity of operative data and the higher prevalence of DM and HT in statin-taking patients, we still observed less NOAF in patients receiving statins. However, there was no significant difference in NOAF between patients taking atorvastatin and rosuvastatin (11.9% - 10.8%). Moreover, this article contains important findings in terms of making a dose comparison for atorvastatin. In the initial analysis for dosage, there were differences significantly affecting NOAF risk, such as age, EuroSCORE II, and CPB times, between patients receiving low and high doses of atorvastatin. Therefore, we performed a PSM analysis to match the baseline parameters that show variations between groups and could pose a risk for NOAF. In the analysis conducted on the newly obtained subgroup with similar characteristics, NOAF was 13.3% in patients receiving atorvastatin at a dose of 20 mg or less, while it was 10.7% in patients receiving more than 20 mg. We detected a partial reduction in the NOAF rate at higher doses, but this reduction was not statistically significant (*p* = 0.61). We could not perform this comparison in the rosuvastatin group due to the insufficient number of patients.

The significant difference observed in atrial fibrillation between statin users and non-users is consistent with many other studies, as well as our previous work [5]. In their meta-analysis, Elgendy et al. reported that statin usage before CABG was associated with a reduction in POAF risk [18], and Khun et al. also reported that preoperative statin usage reduced this risk. By contrast, Zheng and colleagues reported that preoperative statin did not prevent POAF in elective cardiac surgery [3]. Samadifar and colleagues compared atorvastatin and rosuvastatin in CABG patients and reported similar results regarding post-CABG atrial fibrillation [19]. In another study, a benefit was reported with atorvastatin for POAF, but not with rosuvastatin [18]. Abaci and colleagues also reported that the effects of preoperative rosuvastatin or atorvastatin treatment did not differ in preventing postoperative atrial fibrillation [20]. In our cohort, while NOAF was seen less in those taking statins, the NOAF rates were similar between patients taking atorvastatin and rosuvastatin. However, in addition to these studies, in our comparison of well-matched subgroups, we observed similar NOAF rates at different doses. Kourliouros and colleagues, on the other hand, reported that the effect of statin therapy on atrial fibrillation was greater, especially at high doses, and that it did not affect AF after low doses, such as 10 mg [21]. Lee et al. also reported that perioperative use of high-intensity statins was associated with POAF reduction in patients undergoing CABG [22]. The significantly lower occurrence of NOAF in patients taking statins may be due to their known anti-inflammatory, antioxidant, and pleiotropic effects, their positive effects on lipid metabolism, and their prevention of atherosclerosis [19]. The findings in this study support that postoperative NOAF may be seen less with the preoperative use of either atorvastatin or rosuvastatin, but using high doses may not provide a significant additional beneficial effect regarding NOAF. Although the analysis was performed with a well-matched group, the small number of patients in the atorvastatin subgroup may not be sufficient to clarify this issue. Investigating this topic with randomized controlled trials will contribute to the lack of research on this matter.

One of the most important concerns regarding the use of statins is the risk of AKI. In some studies, it has been reported that AKI is more common with rosuvastatin compared to those receiving a placebo, while some studies have suggested that preoperative statins reduce AKI [3]. Shvartz and colleagues compared atorvastatin and rosuvastatin in CABG patients and found no difference in AKI incidence between the groups [13]. He and colleagues reported that perioperative use of rosuvastatin posed a slightly higher risk of AKI compared to atorvastatin [23]. As a result of this study, the postoperative requirement for CRRT was similar in patients receiving atorvastatin and rosuvastatin. Likewise, there was no statistically significant difference in urea, creatinine, sodium, and potassium values in the first postoperative month. The use of atorvastatin in renal dysfunction is more frequently preferred by many surgeons, especially due to its lower excretion from the kidneys compared to rosuvastatin. Indeed, in our patient cohort, although there was no statistical difference, preoperative chronic kidney disease (CKD) was slightly higher in patients receiving atorvastatin compared to others. However, CKD rates and renal function tests were statistically similar across all groups. A study including only CKD patients could better evaluate the effect of statins on renal function in this patient group. Since the primary objective of this research was not to analyze CKD patients, this situation could not be compared. However, current findings support that similar postoperative renal functions can be achieved with the use of atorvastatin and rosuvastatin in CABG patients. Furthermore, similar renal functions were also detected after the use of high- and low-dose atorvastatin.

One of the most common side effects reported with statins is hepatic adverse effects [24]. Differences in the metabolism of atorvastatin and rosuvastatin may also affect postoperative liver functions. However, we found no statistically significant difference between preoperative ALT and AST values and postoperative first-month ALT and AST values in either group. In fact, the latter values were lower compared to preoperative values in both statin users and non-users. Current findings support that preoperative use of both atorvastatin and rosuvastatin has no significant relationship with advanced postoperative liver failure. However, individual risk and benefit evaluation is important regarding statin use, especially in patients with known prior liver failure. Additionally, since drug interactions may be higher due to the hepatic excretion of atorvastatin in particular, caution should be exercised in the postoperative period regarding drugs excreted via the liver.

There are concerns about whether statins increase bleeding due to many antithrombotic mechanisms of action, which were also mentioned in our previous article [5]. Again, due to these antithrombotic effects, a reduction in thrombotic events can be expected. However, in our study, we observed that both bleeding and thrombotic events occurred with similar frequency in patients receiving atorvastatin and rosuvastatin. Postoperative bleeding amounts, the frequency of gastrointestinal bleeding, and postoperative first-month hematocrit and platelet values were similar in both groups. In terms of thrombotic events, CVA rates, PCI requirements, and MACCE rates were also similar in both statin groups. Furthermore, there was no significant difference regarding all these bleeding and thrombotic events in patients using high- and low-dose atorvastatin. Similarly, in terms of mortality and hospital stay, similar results were found in both statin non-users and those taking atorvastatin and rosuvastatin. Again, similar mortality, ICU stay, and hospital stay were found with low and high doses of atorvastatin. Perioperative statin is reported to be associated with a reduction in mortality after cardiac surgery [25]. In another study, it was reported that treatment with atorvastatin or rosuvastatin showed no difference in hospital stay, ICU stay, and 3-month mortality [19]. Other studies have reported that preoperative statin use shortens ICU stay and hospital stay [26,27]. In our study, we determined outcomes with both statin types and different atorvastatin doses. What is interesting is that there was no significant difference between statin users and non-users either, which may be due to the short follow-up duration. In fact, while many postoperative outcomes, such as mortality, MACCE, CVA, and hospital stay, were numerically lower in patients receiving statins, there was no statistically significant difference. The fact that all these patients were CABG patients and often returned to normal sinus rhythm with medical treatment may have resulted in small numerical differences, but not a statistically significant difference in postoperative outcomes. Although the results of this study do not show a significant difference for mortality and other postoperative outcomes in CABG patients, they support the use of at least any statin due to its potential positive effects in terms of NOAF. Although no significant difference was found between statin types, the small number of patients in the rosuvastatin group reduces the statistical power. In addition, because the pleiotropic effects of statins may be related to the duration of use, future studies considering the duration of use would be beneficial.

## 6. Limitations

The current study has several limitations that should be acknowledged. First, it was designed within a retrospective framework, which inherently carries potential for selection bias. Second, a head-to-head comparison was performed only between atorvastatin and rosuvastatin. The primary reason for this was the negligibly low usage of other statin types in our patient population; however, future studies incorporating series where alternative statin regimens are frequently utilized could provide beneficial insights. Third, due to the much more widespread clinical usage of atorvastatin, the initial distribution of patients across the statin groups was not balanced. Furthermore, the exact duration of preoperative statin use was not clearly documented in our records. The differentiation between short- and long-term statin usage could potentially influence these postoperative outcomes. There is a significant numerical difference between the groups due to the differing rates of statin use. The relatively small number of patients and the low incidence of postoperative outcomes, particularly in the rosuvastatin group, significantly reduces the statistical power. Studies with larger and more balanced numbers could help to better investigate this issue. Furthermore, although many preoperative characteristics were similar between patients using and not using statins, the rates of DM and HT differed. The duration of preoperative statin use and patient adherence could not be assessed. A comparison of patients in terms of preoperative cardiovascular optimization, medical surveillance, and concomitant therapies was not possible. Because the patients in the study underwent isolated CABG, echocardiographic findings that could be important for NOAF, such as left atrial size, were not available for all patients and could not be compared. Similarly, comparisons could not be made regarding preoperative medications such as beta-blockers and amiodarone. Finally, although this investigation provides valuable data regarding early postoperative follow-up outcomes, long-term follow-up results are currently lacking.

## 7. Conclusions

In conclusion, the findings of this study demonstrate that the preoperative utilization of either atorvastatin or rosuvastatin was associated with a lower incidence of NOAF in patients undergoing isolated CABG. However, no significant differences were observed between the atorvastatin and rosuvastatin cohorts regarding NOAF and other postoperative outcomes. Furthermore, preoperative atorvastatin administration at doses below and above 20 mg/day showed no statistically significant differences in terms of postoperative outcomes, including NOAF development.

## Figures and Tables

**Table 1:** Comparison of demographic characteristics, comorbid diseases, and operative data of patients among the groups.

	Group A (*n =* 592) Statin −		Group B (*n =* 293) Atorvastatin +		Group C (*n =* 65) Rosuvastatin +		
	Min-Max or *n* (%)	Median (IQR)	Min-Max or *n* (%)	Median (IQR)	Min-Max or *n* (%)	Median (IQR)	*p*
**Demographic data**							
Sex female	108 (18.2)		50 (17.1)		14 (21.5)		0.69
Age (years)	25–86	61.5 (14)	38–84	60 (12)	41–79	60 (9)	0.13
Height (cm)	135–196	170 (11)	140–195	170 (10)	145–182	167 (11)	0.64
Weight (kg)	45–130	80 (17)	48–127	81 (18)	55–116	78 (18)	0.56
**Comorbid diseases**							
Diabetes mellitus	309 (52.2)		181 (61.8)		40 (61.5)		**0.01**
Hypertension	327 (55.2)		204 (69.6)		47 (72.3)		**<0.001**
Chronic kidney disease	39 (6.6)		28 (9.6)		4 (6.2)		0.26
Chronic obstructive pulmonary disease	44 (7.4)		20 (6.8)		3 (4.6)		0.69
Cerebrovascular accident	48 (8.1)		21 (7.2)		3 (4.6)		0.57
Preoperative atrial fibrillation	7 (1.2)		5 (1.7)		1 (1.5)		0.81
Thyroid disorders	47 (7.9)		23 (7.8)		3 (4.6)		0.62
Peripheral artery disease	71 (12)		40 (13.7)		11 (16.9)		0.46
Rheumatic disease	13 (2.2)		8 (2.7)		1 (1.5)		0.80
EuroSCORE II	0.6–19.2	1.3 (1.2)	0.6–12.5	1.3 (1.2)	0.6–10.3	1.4 (1.2)	0.88
**Echocardiographic findings**							
Preoperative EF (%)	20–65	55 (10)	20–65	60 (10)	30–65	55 (10)	0.07
Preoperative TAPSE (mm)	12–35	23 (4)	11–32	23 (5)	15–32	23 (5)	0.40
Postoperative EF (%)	20–66	55 (10)	20–65	55 (10)	30–60	55 (10)	0.29
Postoperative TAPSE (mm)	9–31	16 (3)	10–29	16 (6)	11–26	18 (5)	0.20
**Operative data**							
XCL times (min)	0–217	78 (49)	0–201	71 (43)	0–180	72 (35)	0.18
CPB times (min)	0–407	127 (60)	0–254	119 (51)	0–268	119 (53)	0.10
The number of grafts	1–7	3 (2)	1–6	3 (1)	1–5	3 (2)	0.49
Minimally invasive surgery	12 (2)		9 (3.1)		1 (1.5)		0.56
Left internal thoracic artery usage	532 (89.9)		270 (92.2)		59 (90.8)		0.54
Coronary endarterectomy	28 (4.7)		9 (3.1)		3 (4.6)		0.50
Beating surgery	48 (8.1)		24 (8.2)		3 (4.6)		0.59

IQR: interquartile range, EuroSCORE: the European System for Cardiac Operative Risk Evaluation, EF: ejection fraction, TAPSE: tricuspid annular plane systolic excursion, XCL: cross-clamp, CPB: cardiopulmonary bypass.

**Table 2:** Comparison of laboratory parameters of patients among the groups.

	Group A (*n =* 592) Statin −		Group B (*n =* 293) Atorvastatin +		Group C (*n =* 65) Rosuvastatin +		
	Min-Max or *n* (%)	Median-Mean (IQR)	Min-Max or *n* (%)	Median-Mean (IQR)	Min-Max or *n* (%)	Median-Mean (IQR)	*p*
**Preoperative laboratory parameters**							
WBC (10^9^/L)	2–92	8.5 (3.1)	3.2–18.6	8.4 (2.9)	4.2–20	8.6 (3.8)	0.26
HCT (%)	21–58	41 (6)	24–59	41 (6)	26–51	41 (7)	0.49
Platelets (10^9^/L)	63–939	254.5 (95)	79–586	252 (82)	149–430	251 (84)	0.74
Urea (mg/dL)	7.8–212.9	34.7 (15.3)	8.2–272	33.3 (15.2)	18–75.5	35.9 (15.6)	0.18
Creatinine (mg/dL)	0.35–11.8	0.91 (0.30)	0.5–9.98	0.94 (0.32)	0.52–2.55	0.95 (0.31)	0.21
Sodium (mEq/L)	124–154	139 (4)	125–149	139 (4)	130–145	139 (3)	0.98
Potassium (mEq/L)	2.96–6.37	4.35 (0.58)	2.69–6.0	4.44 (0.61)	3.5–5.78	4.37 (0.65)	0.38
ALT (IU/L)	1–222	18.5 (12)	4–196	19 (12)	7–54	19 (9)	0.90
AST (IU/L)	4–250	20 (12)	5–431	19 (10)	10–58	18 (10)	0.30
LDL-C (mg/dL)	14–377	116 (58)	30–243	87 (51)	31–226	83 (43)	**<0.001**
Total cholesterol (mg/dL)	73–442	191 (70)	51–331	159 (58)	100–302	153 (57)	**<0.001**
HbA1c (mmol/mol)	2.9–16.2	6.2 (2)	3.3–15.1	6.4 (2.4)	5.1–12.1	6.4 (2)	0.28
**Postoperative 1st month laboratory parameters**							
WBC (10^9^/L)	2.6–111	8.1 (3.2)	2.6–23.2	8.3 (3)	4.3–15.7	8.4 (3.8)	0.86
HCT (%)	21–49	35 (6)	24–47	35 (6)	22–38	35 (6)	0.18
Platelets (10^9^/L)	31–892	359.5 (176)	105–817	361.5 (150)	104–665	335 (212)	0.99
Urea (mg/dL)	11.1–199.2	33 (16.3)	10.4–122.2	33.1 (14.4)	18–77.1	40.6 (17.4)	0.41
Creatinine (mg/dL)	0.30–9.41	0.92 (0.30)	0.52–7.17	0.94 (0.31)	0.66–12.3	1.19 (0.47)	0.46
Sodium (mEq/L)	121–149	139 (5)	129–147	138.5 (4)	135–154	142 (4)	0.68
Potassium (mEq/L)	3.2–6	4.5 (0.6)	3.48–5.94	4.6 (0.59)	3–5.75	4.3 (0.88)	0.38
ALT (IU/L)	4–5703	16 (13)	2–353	15 (11)	4–104	14 (8)	0.85
AST (IU/L)	4–7000	15 (8)	7–353	16 (8)	20–138	16 (6)	0.61

IQR: interquartile range, WBC: white blood cells, HCT: hematocrit, ALT: alanine aminotransferase, AST: Aspartate aminotransferase, LDL-C: low-density lipoprotein cholesterol.

**Table 3:** Comparison of postoperative outcomes of patients among the groups.

	Group A (*n =* 592) Statin −		Group B (*n =* 293) Atorvastatin +		Group C (*n =* 65) Rosuvastatin +		
	Min-Max or *n* (%)	Median (IQR)	Min-Max or *n* (%)	Median (IQR)	Min-Max or *n* (%)	Median (IQR)	*p*
Cerebrovascular accident	14 (2.4)		5 (1.7)		0		0.39
Continuous renal replacement therapy	17 (2.9)		6 (2)		0		0.31
NOAF	109 (18.4)		35 (11.9)		7 (10.8)		**0.02**
Deep sternal wound infection	20 (3.4)		12 (4.1)		2 (3.1)		0.84
Gastrointestinal bleeding	2 (0.3)		2 (0.7)		0		0.65
Percutaneous coronary intervention	4 (0.7)		4 (1.4)		1 (1.5)		0.53
MACCE	33 (5.6)		15 (5.1)		1 (1.5)		0.37
Mortality	21 (3.5)		6 (2)		0		0.16
VIS	0–440	0 (5)	0–100	0 (5)	0–20	0 (3)	0.11
Postoperative bleeding (mL)	100–3250	700 (450)	150–2950	700 (445)	225–2200	775 (350)	0.31
Intubation time (hours)	2–672	9 (7)	1–168	9 (7)	4–24	11 (7)	0.07
Intensive care unit stay (days)	1–28	2 (1)	1–22	2 (1)	1–7	2 (1)	0.07
Hospital stay (days)	1–81	7 (4)	2–106	6 (2)	4–69	6 (3)	0.60

IQR: interquartile range, NOAF: new-onset atrial fibrillation, MACCE: major adverse cardiac and cerebrovascular events, VIS: vasoactive inotropic score.

**Table 4:** Comparison of atorvastatin doses across all parameters.

	All Patients (*n =* 293)					Propensity Score Matched (*n =* 150)				
	Group 1 (*n =* 171) Atorvastatin ≤ 20 mg		Group 2 (*n =* 122) Atorvastatin > 20 mg			Group 3 (*n =* 75) Atorvastatin ≤ 20 mg		Group 4 (*n =* 75) Atorvastatin > 20 mg		
	Min-Max or *n* (%)	Median (IQR)	Min-Max or *n* (%)	Media-(IQR)	*p*	Min-Max or *n* (%)	Median (IQR)	Min-Max or *n* (%)	Median-(IQR)	*p*
**Demographic data**										
Sex female	28 (16.4)		22 (18)		0.71	13 (17.3)		14 (18.7)		0.50
Age (years)	38–79	69 (12)	38–84	59 (12)	**0.04**	38–79	59 (12)	38–84	59 (13)	0.83
**Comorbid diseases**										
Diabetes mellitus	109 (63)		72 (59)		0.41	44 (58.7)		46 (61.3)		0.43
Hypertension	118 (69)		86 (70.5)		0.78	48 (64)		55 (73.3)		0.14
Chronic kidney disease	17 (9.9)		11 (9)		0.79	7 (9.3)		5 (6.7)		0.54
COPD	12 (7)		8 (6.6)		0.87	3 (4)		5 (6.7)		0.35
CVA	14 (8.2)		7 (5.7)		0.42	4 (5.3)		5 (6.7)		0.50
Preoperative AF	4 (2.3)		1 (0.8)		0.30	0		1 (1.3)		0.50
EuroSCORE II	0.6–12.5	1.4 (1.5)	0.6–8.1	1.2 (0.8)	**0.048**	0.6–12.5	1.2 (1)	0.6–8.1	1.3 (1.1)	0.77
**Echocardiographic findings**										
Preoperative EF (%)	22–65	60 (15)	20–65	60 (5)	0.23	30–65	60 (5)	20–65	60 (5)	0.48
**Operative data**										
XCL times (min)	0–201	71 (52)	0–144	70 (42)	0.06	0–201	65 (46)	0–140	67 (45)	0.98
CPB times (min)	0–254	122 (54)	0–252	113 (49)	**0.01**	0–230	119 (36)	0–252	119 (50)	0.54
The number of grafts	1–6	3 (1)	1–5	3 (2)	0.15	1–6	3 (1)	1–5	3 (2)	0.44
**Postoperative outcomes**										
CVA	4 (2.3)		1 (0.8)		0.30	1 (1.3)		1 (1.3)		1
CRRT	5 (2.9)		1 (0.8)		0.20	2 (2.7)		1 (1.3)		0.50
NOAF	24 (14)		11 (9)		0.19	10 (13.3)		8 (10.7)		0.61
DSWI	10 (5.8)		2 (1.6)		0.06	7 (6.7)		1 (1.3)		0.10
Gastrointestinal bleeding	1 (0.6)		1 (0.8)		0.66	1 (1.3)		1 (1.3)		1
PCI	1 (0.6)		3 (2.5)		0.19	0		2 (2.7)		0.24
MACCE	11 (6.4)		4 (3.3)		0.22	2 (2.7)		3 (4)		0.50
Mortality	6 (3.5)		0		**0.038**	1 (1.3)		0		0.50
VIS	0–100	0 (5)	0–60	0 (3)	0.20	0–35	0 (3)	0–20	0 (4)	0.82
Postoperative bleeding (mL)	150–2750	700 (450)	200–2950	700 (363)	0.92	150–2700	700 (450)	210–2000	700 (400)	0.55
Intubation time (hours)	3–168	10 (6)	1–84	9 (8)	0.11	3–48	9 (6)	1–84	9 (8)	0.75
ICU stay (days)	1–22	2 (1)	1–9	2 (1)	0.11	1–11	2 (1)	1–8	2 (1)	0.66
Hospital stay (days)	2–106	7 (3)	4–48	6 (3)	0.22	3–104	6 (3)	5–48	6 (3)	0.47

IQR: interquartile range, COPD: chronic obstructive pulmonary disease, CVA: cerebrovascular accident, AF: atrial fibrillation, EuroSCORE: the European System for Cardiac Operative Risk Evaluation, EF: ejection fraction, TAPSE: tricuspid annular plane systolic excursion XCL: cross-clamp, CPB: cardiopulmonary bypass, CRRT: continuous renal replacement therapy, NOAF: new-onset atrial fibrillation, DSWI: deep sternal wound infection, PCI: percutaneous coronary intervention, MACCE: major adverse cardiac and cerebrovascular events, VIS: vasoactive inotropic score, ICU: intensive care unit.

**Table 5:** Multivariate regression analysis for factors affecting new-onset atrial fibrillation.

	Odds Ratio	CI %95	*p*
Gender female	0.814	0.501–1.320	0.40
Age	**1.075**	1.051–1.098	**<0.001**
Diabetes Mellitus	0.742	0.510–1.079	0.11
Hypertension	1.270	0.857–1.884	0.23
Statin usage	**0.609**	0.410–0.906	**0.01**

CI: confidence interval.

## Data Availability

No new data were created or analyzed in this study. Data sharing is not applicable to this article.
